# Prognostic and predictive value of serum carcinoembryonic antigen levels in advanced non-small cell lung cancer patients with epidermal growth factor receptor sensitive mutations and receiving tyrosine kinase inhibitors

**DOI:** 10.18632/oncotarget.20145

**Published:** 2017-08-10

**Authors:** Xin Min Zhao, Jing Zhao, Kai Lin Xing, Si Sun, Zhi Guo Luo, Hui Jie Wang, Jia Lei Wang, Jian Hua Chang, Xiang Hua Wu

**Affiliations:** ^1^ Department of Medical Oncology, Fudan University Shanghai Cancer Center; Department of Oncology, Shanghai Medical College, Fudan University, Shanghai 200032, China; ^2^ Department of Medical Oncology, Tongji University Affiliated Shanghai Pulmonary Hospital, Tongji University Medical School Cancer Institute, Shanghai 200433, China

**Keywords:** non-small cell lung cancer, chemotherapy, epidermal growth factor receptor, tyrosine kinase inhibitors, carcinoembryonic antigen

## Abstract

**Background:**

Despite the widespread use of epidermal growth factor receptor (EGFR) tyrosine kinase inhibitors (TKIs) in advanced or recurrent non-small cell lung cancer (NSCLC), no biomarkers for predicting the efficacy of EGFR-TKIs in patients with EGFR-sensitive mutations have yet been identified. The purpose of our study was to explore the effect of baseline serum tumor markers in stage IIIB/IV NSCLC patients treated with EGFR-TKIs.

**Methods:**

One hundred and seventy-seven patients with stage IIIB/IV NSCLC who harbored EGFR-sensitive mutations and were treated with EGFR-TKIs were retrospectively reviewed. Their levels of CEA, CYFRA 21-1, NSE and CA199 were measured before treatment with EGFR-TKIs.

**Results:**

The response rate for all patients was 54.8%, with a median progression-free survival of 6.6 months and overall survival of 14.8 months. In univariate analyses, patients with CEA levels below the cutoff point (10 ng/ml) had higher RR, better PFS, and better OS than those with CEA levels above 10 ng/mL (RR: 69.2% vs. 43.4%, p*=* 0.001; mPFS: 7.8 months vs. 5.3 months, p=0.029; mOS: 18.8 months vs. 11.8 months, p=0.000). The baseline serum CEA level was an independent factor for RR (odds ratio [OR] =0.322, p=0.001), PFS (hazard ratio [HR] =1.45, p=0.025), and OS (HR=2.133, p=0.000).

**Conclusion:**

Our study suggests that baseline serum CEA levels may play a role in predicting the efficacy of EGFR-TKIs in stage IIIB/IV NSCLC patients with EGFR-sensitive mutations who are treated with EGFR-TKIs.

## INTRODUCTION

Lung cancer is the leading cause of worldwide cancer deaths and is one of the most common cancers in both men and women. It has been estimated to account for over 25% of cancer-related deaths [1]. Approximately 80-85% of lung cancer patients are non-small-cell lung cancer (NSCLC), and approximately 40-50% of these patients are advanced-stage NSCLC. The response rate of first-line chemotherapy is only approximately 30%, and the median overall survival (OS) of patients with metastatic NSCLC is approximately one year [2]. Epidermal growth factor receptor (EGFR) is a proto-oncogene that regulates cell proliferation, metastasis, and angiogenesis [3]. EGFR mutations are known to strongly induce oncogenic potential in NSCLC [4]. In patients with EGFR mutations, it is well-established that classic mutations, such as in-frame deletions in exon 19 and the point mutation L858R in exon 21, are associated with high sensitivity to EGFR tyrosine kinase inhibitors (TKIs). The initial response rate to first-generation EGFR TKIs is approximately 60-80% [5].

Tumor markers (TMs) are widely used in lung cancer management to evaluate the effectiveness of treatments, to monitor for metastases and recurrences after therapy, and to predict the effects of therapy. Carcinoembryonic antigen (CEA), cytokeratin 19 fragments (CYFRA 21-1) and neuron-specific enolase (NSE) are the most commonly used serologic markers for lung cancer management. Besides, CA199 is also an important biomarker for NSCLC.

Of the four TMs, CEA and CYFRA 21-1 are most frequently studied. CEA is a glycoprotein product of the gene CEACAM-5 and is a member of the immunoglobulin superfamily that serves as a cell-adhesion molecule and may also have an effect on innate immunity [6, 7]. CEA is overexpressed in many malignant tumors, including NSCLC, and is readily detected in blood samples, making it valuable for prognosis and follow-up evaluations. High serum CEA levels have been identified as a prognostic factor in both resected NSCLC and in metastatic disease [6, 8–11]. CYFRA 21–1 is a fragment of cytokeratin (CK) 19. Serum CYFRA 21-1 levels have also been demonstrated to be a prognostic factor in patients with metastatic or recurrent NSCLC who receive therapy with EGFR TKIs. Pretreatment serum CYFRA 21–1 levels have been suggested to have prognostic value in patients with lung adenocarcinoma and advanced NSCLC who are receiving surgery [12–14].

However, it is unknown whether these TMs can be used as prognostic factors in patients with advanced lung adenocarcinoma and EGFR-sensitive mutations who are treated with EGFR TKIs. Therefore, in the present study, we investigated the impact of CEA, CYFRA 21-1, NSE and CA199 on the prognosis and prediction of TKI-treated stage IIIB and IV lung adenocarcinoma patients with EGFR-sensitive mutations.

## RESULTS

### Patient characteristics

A total of 177 patients (85 males and 92 females) with a median age of 60 years (range 31-80) were included in this analysis. Eighty-six patients received Erlotinib, and 91 patients were treated with Gefitinib. Ninety-four patients carried an exon 19 deletion mutation, 71 patients had an exon 21 point mutation, 8 patients had an exon 18 point mutation, and 16 patients had an exon 20 point mutation, with 10 patients harboring 2 mutations and 1 patient harboring 3 mutations. Among the 177 evaluable patients, 97 patients exhibited a partial response (PR), 24 patients exhibited stable disease (SD), and 56 patients exhibited progressive disease (PD), with an RR of 54.8%. As of December 15^th^, 2014, 166 patients (93.8%) had progressed from the (p=0.001).disease, with a median PFS (mPFS) of 6.6 months (95% confidence interval [CI] 5.2-8.0 months), and 134 patients (75.7%) had died from any cause, with a median OS (mOS) of 14.8 months (95% CI 12.1-17.5 months). As shown in Table 1, the demographic and clinical characteristics of patients with serum CEA levels below and above the cutoff point were balanced.

**Table 1:** Demographic and clinical characteristics of patients with serum CEA levels

Characteristic	CEA		P value
	≤10 ng/mL (%) (n=78)	>10 ng/mL (%) (n=99)	
Age			
< 60	38 (48.7)	45 (45.5)	0.666
≥60	40 (51.3)	54 (54.5)	
Gender			
female	39 (50.0)	53 (53.5)	0.64
male	39 (50.0)	46 (46.5)	
Smoker			
non-smoker	52 (66.7)	69 (69.7)	0.667
current or ever	26 (33.3)	30 (30.3)	
T stage			
1 or 2	43 (55.1)	45 (45.5)	0.201
3 or 4	35 (44.9)	54 (54.5)	
N stage			
negative	20 (25.6)	17 (17.1)	0.169
positive	58 (74.4)	82 (82.9)	
Pleural effusion			
absent	50 (64.1)	54 (54.5)	0.164
present	27 (35.9)	45 (45.5)	
Lung metastasis			
absent	43 (55.1)	50 (50.5)	0.481
present	34 (44.9)	49 (49.5)	
Brain metastasis			
absent	61 (78.2)	86 (86.9)	0.175
present	16 (20.5)	13 (13.1)	
Bone metastasis			
absent	40 (51.3)	43 (43.4)	0.299
present	38 (48.7)	56 (56.6)	
Liver metastasis			
absent	68 (87.2)	85 (85.9)	0.632
present	9 (12.8)	14(14.1)	

CEA= carcinoembryonic antigen.

### Association between serum TM levels and RR, PFS, OS

As shown in Table 2, the RRs of patients with CEA levels below and above the cutoff point were 69.2% and 43.4%, respectively(p=0.001). The RRs of patients with CYFRA 21-1, NSE, and CA199 levels below the cutoff points were 61.3%, 53.1%, and 58.2%, respectively, while in patients with high CYFRA 21-1, NSE, and CA199 levels, the RRs were 50.5%, 56.8%, and 51.3%, without significant differences (p > 0.05). No demographic or clinical factors were associated with RR.

**Table 2:** Association between factors and RR, PFS and OS

Factors	RR			PFS		OS	
	Non-responder (%)	Responder (%)	P value	(95% CI, months)	P value	(95% CI, months)	P value
CEA							
≤Cutoff point	24 (30.8)	54 (69.2)	0.001	7.8(7.0 – 8.6)	0.029	18.8(13.4 – 24.2)	0.0000
>Cutoff point	56 (56.6)	43 (43.4)		5.3(3.6 – 7.0)		11.8(8.5 – 15.1)	
CYFRA 21							
≤Cutoff point	29 (38.7)	46 (61.3)	0.134	7.8(6.6 – 9.0)	0.230	14.5(10.6 – 18.4)	0.677
>Cutoff point	51 (50.0)	51 (50.0)		5.9(4.5 – 7.3)		16.5(12.0 – 21.0)	
NSE							
≤Cutoff point	38 (46.9)	43 (53.1)	0.674	6.6(4.6 – 8.6)	0.995	14.8(10.6 – 19.0)	0.909
>Cutoff point	42 (43.8)	54 (56.2)		6.9(4.7 – 9.1)		14.9(11.3 – 18.5)	
CA199							
≤Cutoff point	41 (41.8)	57 (58.2)	0.317	7.7(6.1 – 9.3)	0.472	14.9(11.0 – 18.8)	0.306
>Cutoff point	42 (49.4)	54 (50.6)		5.6(4.2 – 7.1)		14.4(9.5 – 19.3)	
Age							
< 60	40 (48.2)	43 (51.8)	0.452	5.5(4.0 – 7.0)	0.300	14.1(10.9 – 17.3)	0.08
≥60	40 (42.6)	54 (57.4)		7.4(5.8 – 9.0)		16.7(12.3 – 21.1)	
Gender							
female	39 (42.4)	53 (57.6)	0.435	7.2(5.8 – 8.6)	0.213	17.5(11.0 – 24.0)	0.151
male	41 (48.2)	44 (54.8)		5.9(4.4 – 7.4)		13.5(10.7 – 16.4)	
Smoker							
non-smoker	52 (43.0)	69 (57.0)	0.382	6.9(5.8 – 8.0)	0.644	16.3(13.4 – 19.2)	0.696
current or ever	28 (50.0)	28 (50.0)		5.5(3.8 – 7.2)		13.4(10.3 – 16.5)	
T stage							
1 or 2	43 (48.9)	45 (51.1)	0.330	6.8(5.1- 8.6)	0.719	16.7(10.7- 22.8)	0.259
3 or 4	37 (41.6)	52 (58.4)		6.6(4.7 – 8.6)		14.1(10.6 – 17.7)	
N stage							
negative	19 (51.4)	18 (48.6)	0.398	7.5(6.0 – 9.0)	0.752	17.0(11.7 – 22.2)	0.404
positive	61 (43.6)	79 (56.4)		6.6(5.1 – 8.1)		14.8(11.9 – 17.7)	
Pleural effusion							
none	49 (47.1)	55 (52.9)	0.595	7.5(6.3 – 8.7)	0.109	18.6(13.6 – 23.5)	0.009
present	31 (43.1)	41 (56.9)		5.5(4.8 – 6.2)		11.5(9.8– 13.2)	
Lung metastasis							
none	43 (46.2)	50 (53.8)	0.825	6.8(5.1 – 8.6)	0.416	14.9(9.7 – 20.1)	0.566
present	37 (44.6)	46 (55.4)		6.6(4.8 – 8.5)		14.8(11.7 – 17.9)	
Brain metastasis							
none	66 (44.9)	81 (55.1)	0.738	7.1(5.5 – 8.6)	0.078	14.8(11.8 – 17.8)	0.125
present	14 (48.3)	15 (51.7)		5.7(3.4 – 8.0)		14.8(8.1 –21.4)	
Bone metastasis							
none	37 (44.6)	46 (55.4)	0.876	6.6(5.0 – 8.2)	0.985	14.9(9.7 – 20.0)	0.387
present	43 (45.7)	51 (54.3)		6.9(4.5 – 9.3)		14.8(11.4 – 18.1)	
Liver metastasis							
none	69 (45.1)	84 (54.9)	0.806	6.6(5.0 – 8.2)	0.957	14.8(11.9 – 17.7)	0.230
present	11 (47.8)	12 (52.2)		6.9(4.8 – 9.0)		14.4(10.2 – 18.6)	

RR= response rate ; PFS= progression-free survival; OS= overall survival; CI=confidence interval.

### Serum CEA level is an independent predictive factor for PFS and OS in patients with EGFR mutations

**Figure 1:**
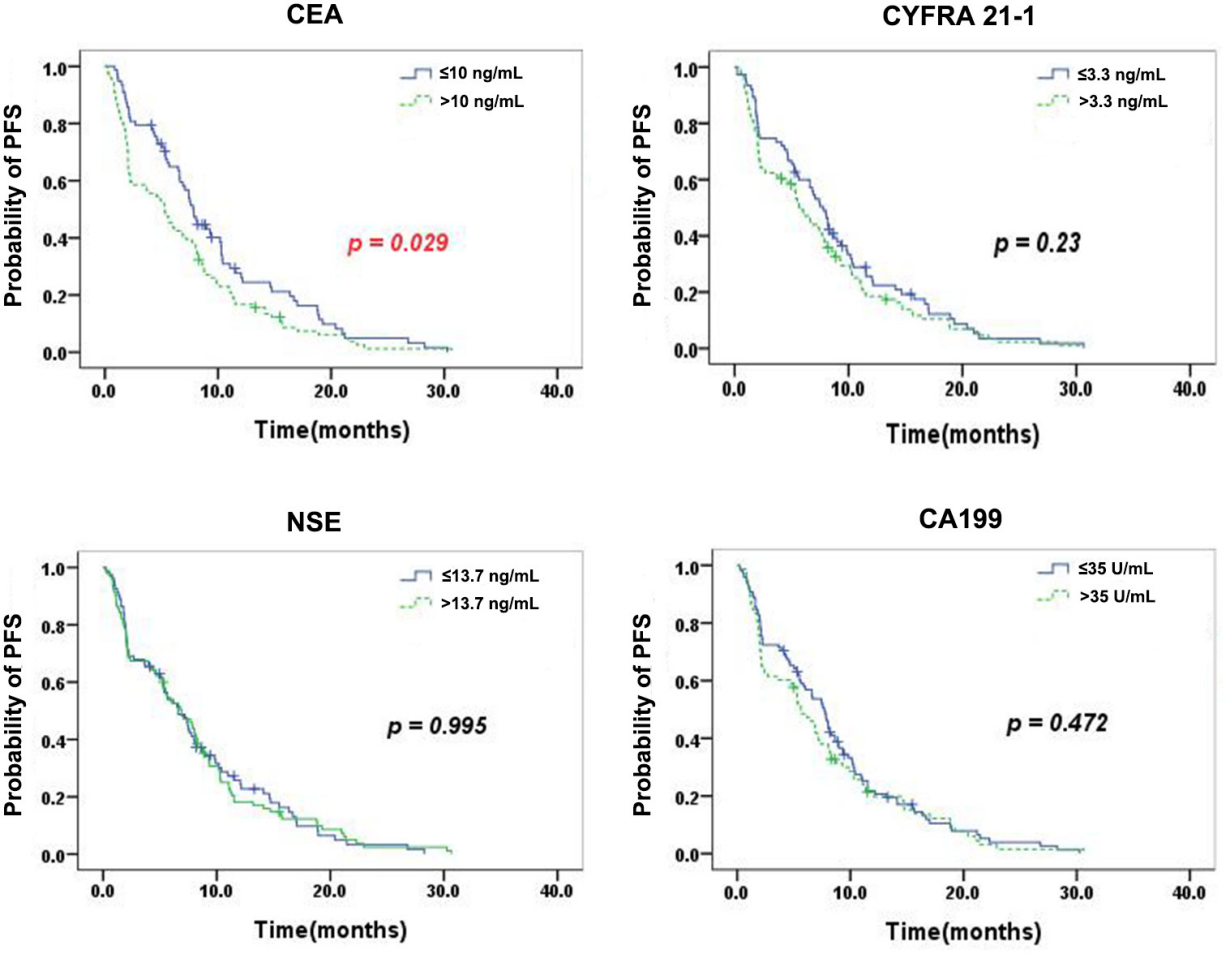
Kaplan–Meier curves for PFS according to the serum levels of single TMs Patients with serum CEA levels below 10 ng/mL displayed a significantly improved mPFS for patients with higher CEA (p= 0.029). As for CYFRA 21-1, NSE, and CA199, there were no significant differences in mPFS between patients with low and elevated TM levels.

Kaplan-Meier curves for PFS according to the serum levels of single TMs are shown in Figure 1. Patients with serum CEA levels below 10 ng/mL displayed a significantly improved mPFS of 7.8 months (95% CI, 7.0-8.6), vs. 5.3 months (95% CI, 3.6-7.0) for patients with higher CEA (p= 0.029). As for CYFRA 21-1, NSE, and CA199, there were no significant differences in mPFS between patients with low and elevated TM levels (CYFRA 21-1: 7.8 months [95% CI, 6.6-9.0] vs. 5.9 months [95% CI, 4.5-7.3], p=0.23; NSE: 6.6 months [95% CI, 4.6-8.6] vs. 6.9 months [95% CI, 4.7-9.1], p=0.995; CA199: 7.7 months [95% CI, 6.1-9.3] vs. 5.6 months [95% CI, 4.2-7.1], p= 0.472).

**Figure 2:**
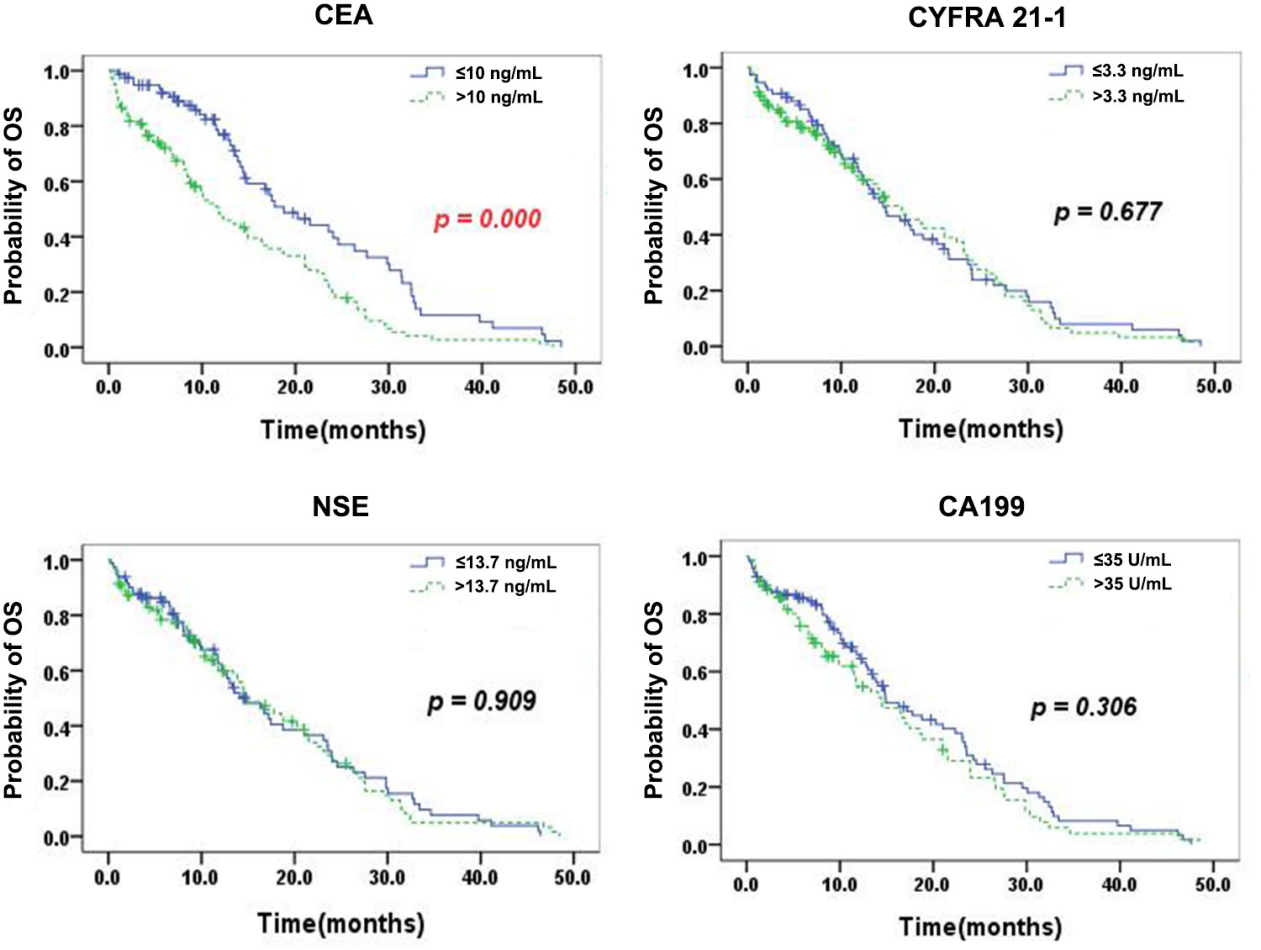
Kaplan–Meier curves for OS according to the serum levels of single TMs Patients with normal serum CEA exhibited a significantly longer mOS than those with elevated CEA (p=0.000). No significant differences in mOS between patients with normal and high TM levels were observed (CYFRA 21-1, NSE, and CA199).

Figure 2 shows the Kaplan–Meier curves for OS according to the serum levels of single TMs. Patients with normal serum CEA exhibited a significantly longer mOS than those with elevated CEA (18.8 months [95% CI, 13.4 – 24.2] vs. 11.8 months [95% CI, 6.9 – 16.7], p=0.000). No significant differences in mOS between patients with normal and high TM levels were observed (CYFRA 21-1: 14.5 months [95% CI, 10.6 -18.4] vs. 16.5 months [95% CI, 12.0-21.0], p=0.677; NSE: 14.8 months [95% CI, 10.6-19.0] vs. 14.9 months [95% CI, 11.3-18.5], p=0.909; CA199: 14.9 months [95% CI, 11.0-18.8] vs. 14.4 months [95% CI, 9.5-19.3], p =0.306.

Multivariate analysis with a binary logistic regression model revealed that the serum CEA level before EGFR TKI treatment was an independent predictive factor for RR (odds ratio [OR]=0.322, 95% CI, 0.166-0.625, p=0.001;. Cox regression multivariate analysis demonstrated that CEA was independent predictive factor for PFS (hazard ratio [HR]=1.45, 95% CI, 1.047-2.008, p=0.025) and for OS (HR = 2.133, 95% CI, 1.444-3.151, p = 0.000) (Table 3).

**Table 3:** Multivariate analysis for RR, PFS and OS

Variables	RR			PFS			OS		
	HR	95% CI	P value	HR	95% CI	P value	HR	95% CI	P value
CEA (>10 ng/mL vs≤10 ng/mL)	0.322	0.166 – 0.625	0.001	1.450	1.047 – 2.008	0.025	2.133	1.444 – 3.151	0.000
CYFRA 21-1 (> 3.3 ng/ml vs≤13.3g/ml)	0.595	0.294 – 1.201	0.147	1.217	0.871 – 1.702	0.250	0.864	0.583 – 1.282	0.468
NSE (>13.7 ng/mL vs≤13.3g/ml)	1.724	0.861 – 3.452	0.124	0.838	0.598 – 1.173	0.302	0.896	0.610 – 1.316	0.576
CA199 (>35 U/ml vs≤35 U/ml)	0.788	0.416 – 1.492	0.464	1.108	0.807 – 1.521	0.527	1.277	0.898 – 1.816	0.174

RR= response rate ; PFS= progression-free survival; OS= overall survival; HR=hazards ratio; CI=confidence interval.

## DISCUSSION

In our study, we demonstrate that serum CEA levels are independent prognostic factors in TKI-treated stage IIIB and IV lung adenocarcinoma patients. We also show that a serum CEA level of less than 10 ng/ml was a predictor of favorable outcomes in advanced lung adenocarcinoma patients with EGFR-sensitive mutations (exon 19 deletion and L858R). In all patients, OS values were worse in conjunction with elevated values of serum tumor markers, but only in the case of CEA did this association reach statistical significance. Our study provide evidence that CEA levels may play a role in predicting the efficacy of EGFR TKIs, which is of great value for the selection of appropriate therapies.

The major strength of our study is that we chose a specific population. We excluded wild-type EGFR and T790M lung adenocarcinoma patients who were resistant to 1^st^-generation EGFR-TKIs such as Gefitinib and Erlotinib. And we included patients from 2009 and used AJCC lung cancer staging edition version 7, the most recent version, to classify patients with malignant pleural effusion as stage IIIB. The prognoses of these patients were as poor as those classified as stage IV, due to more accurate prognostic factor analyses. To avoid interfering factors, we used a new staging system [16]. Besides, all patients received platinum-based chemotherapy as a first-line treatment and received EGFR-TKIs as a second- or third-line therapy, thus avoiding the interference of different lines of therapy. We did not analyze patients treated with first-line EGFR-TKIs, because in 2009, patients with EGFR exon 19 deletion mutations or L858R were not routinely treated with TKIs as a first-line therapy [17].

CEA is a very nonspecific tumor biomarker that shows elevated expression in various solid tumors. In lung cancer, inconsistent results have been published concerning the prognostic value of baseline CEA levels. In the Tomita et al [18] study of 291 stage I-III NSCLC patients, CEA was found to be a significant prognostic factor in patients with normal and high serum CEA levels, predicting 71.52% and 48.41% of cases, respectively (p<0.0001). In contrast to our study, Tomita et al recruited operable NSCLC patients, and after surgery all patients received adjuvant chemotherapy without TKIs. Moreover, their study did not determine EGFR status. Cedres et al [8] conducted a study to detect baseline CEA levels in 277 advanced-stage NSCLC patients and found that high baseline levels of tumor markers are correlated with worse survival in stage III-IV NSCLC patients. However, Cedres et al recruited patients with squamous cell carcinoma, large cell carcinoma, and adenocarcinoma, and they did not classify EGFR status [10]. In the Moro et al [12] study of 105 all-stage NSCLC patients, CEA was found to be a significant negative prognostic factor, but their study examined all-stage patients with different subtypes and used an older staging system. However, in studies from Ardizzoni et al. and Kulpa et al. of 107 patients with advanced-stage disease and of 200 patients of all stages, respectively, CEA was not found to be a prognostic factor for survival [19, 20].

In our study, the cutoff level for CEA was 10 ng/ml and patients with CEA levels higher than 10 ng/ml had greater PFS and OS values. The cutoff level for CEA ranged between 2.5 ng/ml and 40 ng/ml in different studies [21]. These varying cutoff levels are likely due to different techniques and routines used in different testing centers. The results of some studies with cutoff values above 10 ng/ml were quite similar to ours, showing the prognostic value of serum CEA levels and possibly demonstrating that the use of various tests to find the cutoff point provides the best description of the true value.

There is also some limitations in our study. Some biases were unavoidable due to chose two EGFR TKI-sensitive mutations in the study, EGFR exon 19 deletion and L858R, which are both sensitive mutations but differ in their degree of sensitivity [22]. Additionally, our study sample size was small, as the patients were drawn from a single treatment center. These inconsistencies should be resolved by further external validations, preferably across multiple centers.

## CONCLUSION

In this series of patients with advanced lung adenocarcinoma and EGFR-sensitive mutations who were treated with EGFR TKIs, CEA could be used as a significant predictive and prognostic tumor marker when selected 10 ng/ml as cut off point of pretreatment serum CEA levels.

## MATERIALS AND METHODS

### Patients

This study was conducted in the Department of Medical Oncology at Fudan University Shanghai Cancer Center between September 2009 and August 2013. A total of 177 patients were deemed eligible on the basis of the following criteria: (1) histopathologically confirmed, locally advanced or metastatic pulmonary adenocarcinoma harboring EGFR exon 18-21 mutations, except for those with an exon 20 mutation only; (2) receiving platinum-based chemotherapy as a first-line therapy; (3) receiving EGFR TKIs as a second- or third-line therapy; (4) available tumor marker (TM) data before treatment with EGFR TKIs; and (5) available follow-up data. Demographic and clinical characteristics, including gender, age at diagnosis of lung cancer or recurrence (< 60 or ≥ 60 years), smoking status, TNM staging status, and presence of metastatic organs at the time of treatment with TKIs were collected. Baseline circulating TMs were analyzed as potential predictive and prognostic factors. This study was approved by the Ethics Committee of Fudan University prior to commencing.

### Treatment and evaluation of therapeutic efficacy

All 177 patients received platinum-based chemotherapy as a first-line therapy and EGFR TKIs as a second- or third-line therapy for at least one month. Gefitinib was given at a dose of 250 mg qd, and Erlotinib was given at a dose of 150 mg qd. The numbers of metastatic sites at the diagnosis were counted on computed tomographic images, brain magnetic resonance images, whole body bone scans. The tumor response was assessed based on the Response Evaluation Criteria in Solid Tumors (RECIST) version 1.1 [15].

### Tumor marker detection

The serum levels of TMs, including CEA, CYFRA 21-1, NSE, and CA199, were tested before EGFR TKI treatment using a chemiluminescence immunoassay. Four TMs were tested in a single laboratory and the cut-off values for each marker were 10 ng/mL for CEA, 3.3 ng/mL for CYFRA-21, 13.7 ng/mL for NSE, and 35 U/ml for CA19-9. Baseline serum levels of TMs were considered available if they were tested within one week before EGFR TKI treatment began.

### Statistical analysis

Statistically significant differences in categorical variables between predictive factors and response rate (RR) were analyzed Pearson’s χ2 test or Fisher’s exact test as appropriate. Progression-free survival (PFS) is defined as the time from the date of starting EGFR-TKI treatment to the date of disease progression or death from any cause, if a patient died earlier; surviving patients without progression were evaluated at their most recent follow-up. Overall survival (OS) was calculated from the start of the first-line treatment of advanced disease to the date of death or the last follow-up. PFS and OS were estimated by the Kaplan-Meier method. The association between predictive factors and PFS or OS was explored using a log-rank test. The Cox proportional hazards regression model was used to determine the hazards ratios (HR) and 95% confidence intervals (CIs) in the univariate and multivariate survival analyses. Two-sided values of P <0.05 were considered statistically significant.

## Abbreviations

NSCLC: non-small-cell lung cancer
EGFR: epidermal growth factor receptor
TKIs: tyrosine kinase inhibitors
TMs: tumor markers
CEA: carcinoembryonic antigen
PFS: progression-free survival
RR: response rate
OS: overall survival
